# Assessing metabolic alterations and cancer biomarkers in athymic nude mice implanted with NSCLC spheroids

**DOI:** 10.1007/s11306-025-02274-8

**Published:** 2025-06-11

**Authors:** Charity Mabela, Chrisna Gouws, Shayne Mason, Wihan Pheiffer

**Affiliations:** 1 DSI/NWU Preclinical Drug Development Platform, Faculty of Health Sciences, North-West University (Potchefstroom Campus), Private Bag X6001, Potchefstroom, 2520 South Africa; 2 Centre of Excellence for Pharmaceutical Sciences (PharmaCen), Faculty of Health Sciences, North-West University (Potchefstroom Campus), Private Bag X6001, Potchefstroom, 2520 South Africa; 3 Human Metabolomics, Faculty of Natural and Agricultural Sciences, North-West University (Potchefstroom Campus), Private Bag X6001, Potchefstroom, 2520 South Africa

**Keywords:** A549 spheroids, Cancer biomarkers, Metabolomics, Miniaturised NMR, spheroid-derived xenograft

## Abstract

**Background:**

Preclinical rodent models are vital in cancer research, and enhancing their relevance reduces animal use.

**Aim:**

The aim of this pilot study was to determine if A549 lung cancer spheroids implanted into athymic nude mice will change the serum metabolite profile and express serum cancer biomarkers.

**Method:**

Spheroid non-small cell carcinoma tumours were implanted into athymic nude mice. ^1^H-NMR metabolomics was used to evaluate the serum metabolome. Specific cancer biomarkers were determined using commercial kits.

**Results:**

Tumours produced significant levels of selected serum cancer biomarkers, and the implanted tumours had changed the serum metabolite profiles—mimicking human lung cancer profiles—which were significantly different from the non-cancer control. Histological analysis confirmed characteristics of solid adenocarcinomas.

**Conclusion:**

The A549 spheroid-derived xenograft model demonstrates promise as a feasible and physiologically relevant tool for non-small cell lung cancer research.

## Introduction

Lung cancer is a global health concern that affects millions of people, with non-small cell lung cancer (NSCLC) being the most prevalent type (Thandra et al., 2021). To combat this disease, extensive research is being conducted in preclinical settings to better understand its complexities and develop more effective detection and treatment strategies (Arnal-Estapé et al., 2021).

Among the current in vitro models, three-dimensional (3D) spheroids have gained popularity as self-assembled cell agglomerates resembling a solid tumour structure (Pinto et al., 2020). Spheroids are considered physiologically relevant models because they replicate the 3D organisation and cellular interactions within solid tumours (Shie et al., 2023). Spheroids have some limitations, such as the absence of a functioning circulatory system, which is important in tumour biology research for studying long-term growth and the observed metastatic potential (Kim et al., 2022). This limitation complicates the investigation of tumour responses to treatments reliant on vascular interactions and drug delivery systems (Monleón-Guinot et al., 2023). Consequently, researchers often use complimentary techniques known as xenografts, which is the subcutaneous or orthotopic inoculation of cells in animals, particularly immunocompromised rodents (Guerin et al., 2020).

Subcutaneous xenografts are valuable tools for drug efficacy and toxicity evaluations after initial in vitro screening (Pan et al., 2020; Choi et al., 2021; Jun et al., 2023). The subcutaneous cell suspension inoculation A549 NSCLC xenograft model is simple and well-documented for convenient tumour monitoring and measurement (Xu & Prestwich, 2010).

However, implantation of spheroids into rodents as models has shown to be more successful than using a cell suspension (Szade et al., 2016; Huang et al., 2020; Choi et al., 2021; Bastian et al., 2024). Integrating 3D cell cultures and rodent models could remove some of the limitations of current models (Guerin et al., 2020; Bastian et al., 2024). The gap in knowledge of 3D or spheroid-derived xenograft (SDX) in terms of growth rates, biochemistry and metabolite profiles is a new field to be studied. Lung cancer biomarkers and metabolites in subcutaneous xenograft models and metabolic pathway alterations offer insights into tumour metabolism and therapeutic targets (Szade et al., 2016). Biomarkers like carcinoembryonic antigen (CEA) and carbohydrate antigen 125 (CA-125) are crucial in various stages of the disease (Saad et al., 2022). These biomarkers aid in determining disease progression, evaluating treatment efficacy, and predicting patient outcomes (Zhang et al., 2015). Additionally, analysing alterations in metabolites linked to lung cancer can show alterations and changes at the molecular level to better understand the disease (Mason et al., 2018). This insight aids in early detection, comprehension of tumour biology, identification of therapeutic targets, and monitoring treatment responses, thereby contributing to improved patient care and outcome (Guo et al., 2021).

An A549 NSCLC spheroid model was previously developed and characterised, and was shown to be metabolically stable and mature after 9 days in culture (Mabela et al., 2024). The spheroids were homogenously round and compact, and histological assessment showed that they produced mucin, which demonstrated the model’s physiological relevance.

This pilot study aimed to determine if A549 spheroids implanted into a mouse model will express cancer-specific biomarkers and changes in metabolic pathways, relative to a non-cancer control. This can be a novel approach for xenograft studies to provide insights into cancer biology, cancer metabolite profiling, and pharmacometabolomics.

## Materials and methods

### Cultivation and maintenance of A549 spheroids in a clinostar™ system

The A549 non-small cell lung adenocarcinoma cell line (ATCC^®^ CCL-185™) was maintained in 10% foetal bovine serum supplemented Ham’s F-12 K medium at standard conditions (Cooper et al., 2016). Cells were cultured to 80–90% confluency and harvested with 0.25% Trypsin-Versene. As previously established, 2.5 × 10^6^ cells were placed into a CelVivo ClinoReactor™ with supplemented media and 0.06% v/v ascorbic acid (Mabela et al., 2024). The ClinoReactor™ was placed in a CelVivo ClinoStar™ system at standard culturing conditions to form spheroids (van Niekerk et al., 2023). The rotation speed was monitored to accommodate spheroid growth by maintaining a microgravity environment (Wrzesinski et al., 2021; Mabela et al., 2024). Culture media was exchanged every second day, and spheroids were harvested at a size range of 0.6 to 1 mm in diameter, which was approximately 16 days of 3D culture.

### Animals and spheroid implantation

Fifteen (*N* = 15) specific pathogen-free male and female athymic nude mice, aged 6 weeks, were bred and housed at the DSI/NWU Preclinical Drug Development Platform (PCDDP) Vivarium. Animal welfare, monitoring and experiments fully complied with guidelines approved by the NWU-AnimCareREC (NWU-00437-21-A5). One spheroid (approximately 5 µg protein/spheroid) (Mabela et al., 2024) was suspended in 200 µL 1:1 F12K: Matrigel media and subcutaneously implanted into the right flank of ten (*n* = 10) mice using a 25-gauge needle (SDX group). The remaining five (*n* = 5) mice served as a negative non-cancer control. Serum and tissue samples were collected after euthanasia on day 25 post-implantation.

### Histological assessment

The excised tumours were prepared according to standard procedures (Van der Merwe et al., 2022). In short, fixed and dehydrated tumours were paraffin-embedded, sectioned, dewaxed, and stained with haematoxylin and eosin (H&E). The sections were imaged using an Image XpressPICO™ (Molecular Devices) imaging system.

### Analysis of serum cancer biomarkers and metabolomics

ELISA kits from Elabsciences^®^ (E-EL-M0232) and Cloud-Clone Corp^®^ (SEA154MU) were used to determine the serum levels of carcinoembryonic antigen (CEA) and carbohydrate antigen 125 (CA-125), respectively. All serum samples and standards were processed in triplicate and analysed according to the manufacturer’s instructions.

The miniaturised proton nuclear magnetic resonance (^1^H-NMR) spectroscopy protocol, as described by Mason et al. (2018), was used to detect changes in untargeted serum metabolites of the SDX group compared with the non-cancer control. In short, all serum samples were filtered by centrifugation to remove macromolecules and proteins—to prevent interference with small molecule analysis. The centrifuge filters were prerinsed 5 times to remove glycerol from the filtrate; this was done by adding 100 µL of HPLC purity water and centrifuging at 3000 x *g* for 5 min at 25 ºC. Afterwards, 100 µL of serum samples were pipetted into the prerinsed centrifuge filter unit and centrifuged at 3000 x g for 30 min. Then, 54 µL of filtered serum was aspirated using the eVol^®^ NMR digital syringe and placed in a 2 mm NMR tube. Then, 6 µL of NMR buffer solution (potassium phosphate buffer at pH 7.4, with 5.8 mM internal standard trimethylsilyl propanoic acid in deuterium oxide) was aspirated, added to the serum, and mixed gently to ensure homogeneity. Each NMR tube was loaded onto a Sample Xpress autosampler for analysis on a 500 MHz NMR spectrometer with a 5 mm triple-resonance inverse TXI probe at room temperature. The NMR acquisition parameters were the same as described by Mason et al. (2018).

### Statistical analysis

Data are presented as mean ± SD. Shapiro-Wilk test was used to test for normality. The non-parametric Mann-Whitney U test was used to compare the serum biomarkers and metabolite data between the SDX and control groups. Differences were considered significant when the *p*-value was less than 0.05. Statistical analysis was done using GraphPad Prism 9.4.1.

## Results and discussion

### A549 spheroid-derived xenografts

**Fig. 1:**
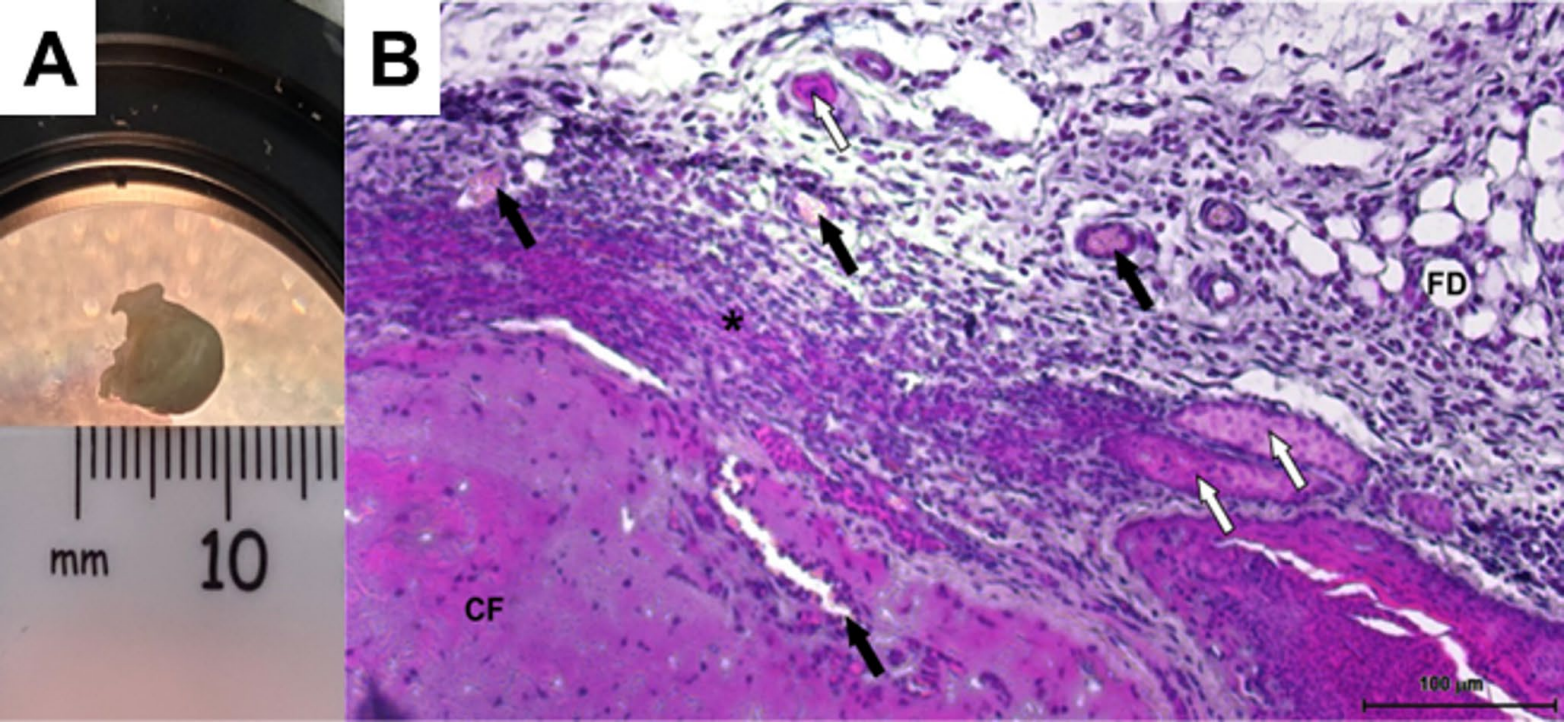
A549 excised tumour (SDX animal #6) after 25 days. **A** H&E staining of excised tumours from spheroid derived xenografts under 10x magnification. **B** Black arrows indicate vascularisation, white arrows indicate glandular structures, asterisk shows compact cell packing. *CF* central fibrosis, *FD*  fat deposit, Scale bar = 100 μm

A549 spheroids (0.71 ± 0.16 mm) were implanted in ten mice, of which four animals developed palpable, measurable tumours after 25 days (Table 1). Huang et al. (2020) reiterate that there is a large variation between animals in terms of tumour growth. The resulting tumours in this study had a mean volume of 16.3 ± 10.97 mm³, which was a mean fold increase of 108 relative to the implanted size. The largest volume increase (215.4-fold) was for the largest implanted spheroid (Table 1; Fig. 1A).

**Table 1:** Summary of implanted A549 spheroids in athymic nude mice (giving actual implanted sizes (mm) and volumes (mm^3^), the tumour take rates (%), excised tumour volumes (mm^3^), and tumour volume fold increase), compared to subcutaneous injected A549 xenografts (giving inoculation density, inoculation volume, the tumour take rates (%), excised tumour volumes (mm^3^))

Spheroid-derived xenograft					
Animal no	Implanted spheroid (mm)	Volume of spheroid (mm^3^)	Visible and palpable (yes/no)	Excised tumour volume (mm^3^)	Fold increase
1	0.6	0.11	No	–	–-
2	0.6	0.11	No	–	–-
3	0.8	0.27	No	–	–
4	0.9	0.38	**Yes**	**23**	**60.5**
5	0.6	0.11	No	–	–
6	1	0.52	**Yes**	**112**	**215.4**
7	0.6	0.11	**Yes**	**10**	**90.1**
8	0.5	0.07	No	–	–
9	0.8	0.27	**Yes**	**18**	**66.7**
10	0.7	0.18	No	–	–

Notable results are given in bold

The growth rates of the A549 spheroids indicate promising growth potential in terms of the fold increases reported over the 25 days. The excised tumour volumes were considerably larger than another A549 SDX model, with lower volume increases for similar-sized spheroids — reporting tumour volumes of ± 0.25 mm^3^ after 25 days (Huang et al., 2020). These findings imply that implanting larger spheroids may result in proportionally larger tumours, suggesting scalable growth potential in this model. This is corroborated by tumour fragments (1–3 mm^3^) of solid tumours (grown from loose cells) implanted into mice (Lu et al., 2009; Xu & Prestwich, 2010; Szade et al., 2016).

### Histology of the excised tumour

The excised tumours exhibited common adenocarcinoma characteristics (Zheng, 2016). These included cuboidal epithelial glandular structures (white arrows), sheets of patternless eosinophilic tumour cells on fibroblastic stroma (asterisk), and a central fibrosis area (CF) (Fig. 1B). The implanted A549 spheroids developed vascularisation (black arrow) and fatty deposits (FD) (Fig. 1B).

### Cancer biomarkers and serum metabolites in spheroid-derived xenograft

Even though only a few tumours were palpable, all inoculated animals were grouped together, namely the spheroid-derived xenograft group. The main aim of this pilot study was to determine if implanted 3D cultures would change the metabolic profile in the rodents, as well as express cancer-specific biomarkers. Elevated CEA and CA-125 levels have been associated with lung adenocarcinoma (Xu et al., 2016). Although CEA alone is not used to diagnose cancer, monitoring its levels in conjunction with other tests aids in tracking treatment responses and disease progression (Zhang et al., 2015). CA-125 is a marker protein for the diagnosis and management of cancers, including lung cancer (Wu et al., 2018).

**Fig. 2:**
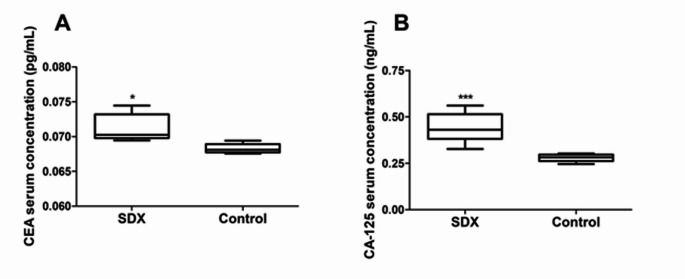
Cancer biomarkers. **A** Serum carcinoembryonic antigen (CEA) concentration (pg/mL ± SD) and **B** serum carbohydrate antigen 125 (CA-125) concentrations (ng/mL ± SD), quantified in the serum of A549 spheroid derived xenograft (SDX) athymic nude mice (n = 10) 25 days post implantation relative to the non-cancer control (n = 5). Asterisk (*) indicates statistical differences compared to the control: * = p <0.05 and *** = p < 0.001

The concentration CEA was significantly greater (*p =* 0.0127) in the SDX groups compared to the control (Fig. 2A). Similarly, the serum concentration of CA-125 was also significantly greater (*p =* 0.0007) in the SDX group compared to the control (Fig. 2B). There were no significant differences within the SDX group for either CEA (*p* = 0.76) or CA-125 (*p* = 0.26) when comparing the levels between the animals with palpable tumours and those without. The highest biomarker levels corresponded to the largest tumour volumes. This relationship between elevated concentrations and tumour growth (Table 1) highlights the potential of the model for tracking lung cancer progression. Thus, the A549 SDX model group appropriately expresses lung cancer biomarkers in the serum.

**Fig. 3:**
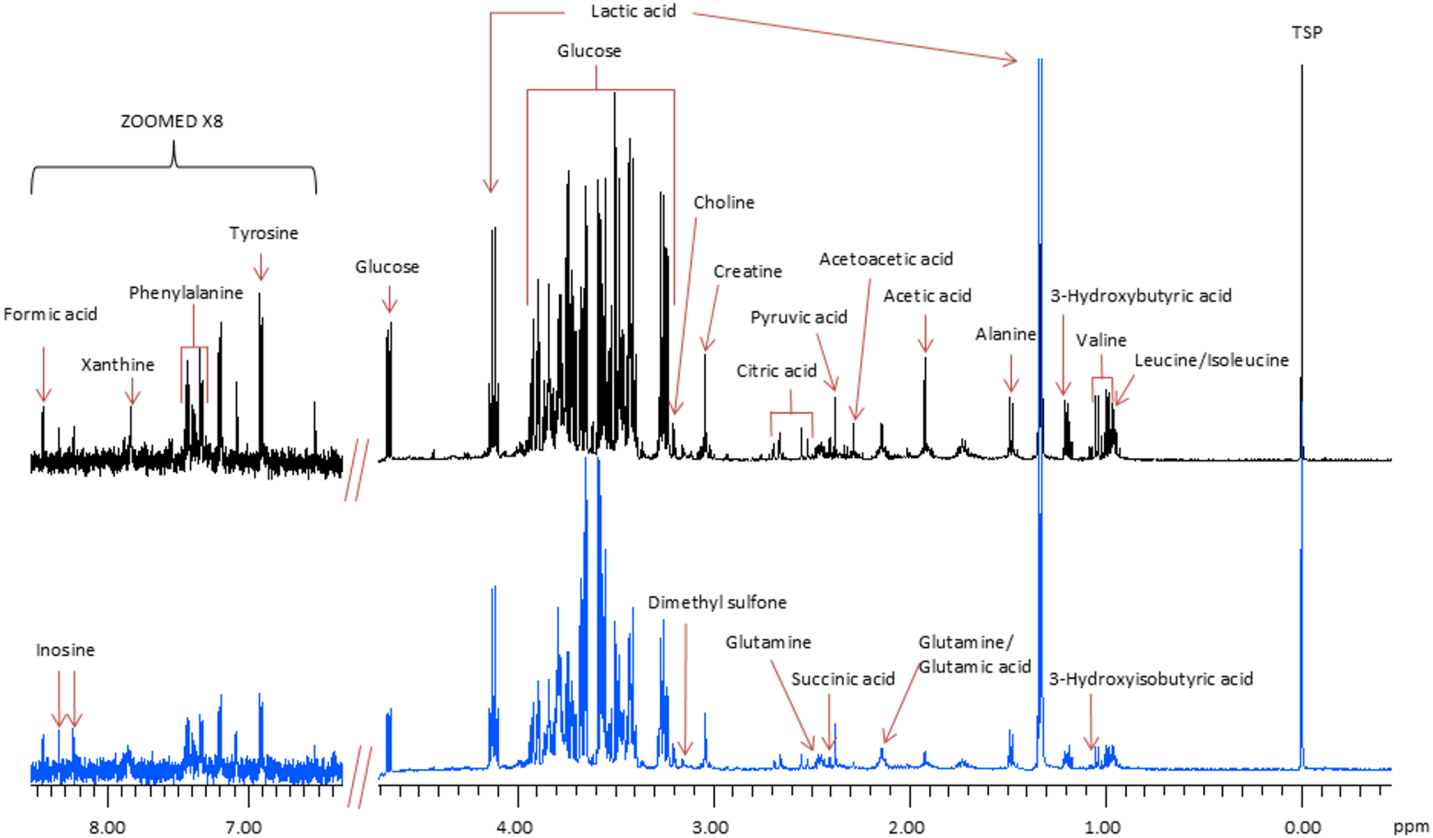
Representative ^1^H-NMR spectra of serum from the control group (bottom; blue) compared with the experimental group (top; black), with the 26 metabolites indicated

Twenty-six metabolites were identified in the serum, of which only eight significantly differed between the groups (Fig. 3; Table 2). The mean concentrations of isoleucine, leucine, valine, 3-hydroxybutyric acid, creatine, pyruvate, glutamine, acetoacetic acid, and phenylalanine in the SDX serum were significantly greater (*p* < 0.05) than the levels in the control mice (Table 2). There were no significant differences within the SDX group between animals with palpable tumours and those without (data not shown).

**Table 2:** A summary of the detected metabolites and their concentrations (µmol/L ± SD) quantified in the serum of the A549 spheroid derived xenograft (SDX) mice (n = 10) compared to the control non-cancer athymic nude mice (n = 5)

Detected serum metabolites	A549 Spheroid derived xenograft serum concentration (µmol/L)	Control non xenograft mice serum concentrations (µmol/L)
Isoleucine	161.6 ± 58.1**	75.1 ± 27.9
Leucine	264.7 ± 97.3**	139.5 ± 59.7
Valine	319.5 ± 139.3**	162.7 ± 68.6
3-Hydroxyisobutyric acid	86.5 ± 32.3**	46.2 ± 13.8
Lactic acid	10558.0 ± 4231.3	8948.1 ± 3100.9
Alanine	471.2 ± 159.6	282.0 ± 105.1
3-Hydroxybutyric acid	268.2 ± 124.1	236.6 ± 63.1
Acetic acid	237.1 ± 177.5	126.8 ± 93.4
Glutamic acid	248.3 ± 96.4	131.9 ± 43.3
Glutamine	743.7 ± 246.3*	479.2 ± 111.5
Acetoacetic acid	61.7 ± 39.4*	28.3 ± 3.8
Pyruvic acid	269.1 ± 111.9*	135.1 ± 81.3
Succinic acid	62.2 ± 25.3	42.7 ± 7.8
Citric acid	450.1 ± 156.5	287.0 ± 83.4
Creatine	331.4 ± 137.4*	196.7 ± 54.6
Dimethyl sulfone	29.5 ± 10.8	17.6 ± 4.1
Choline	66.7 ± 37.5	40.6 ± 9.2
Total glucose	2382.3 ± 1093.8	1727.2 ± 820.5
Tyrosine	96.5 ± 37.8	57.9 ± 33.2
Phenylalanine	122.7 ± 41.5*	52.6 ± 26.8
Xanthine	4.1 ± 8.7	5.4 ± 12.2
Inosine	11.8 ± 18.9	ND
Formic acid	22.8 ± 30.1	3.9 ± 3.6

Significant results are reported in bold

Asterisk (*) indicates statistical differences compared to the control: * = p < 0.05 and *** = p < 0.005

*ND*  not detected

The Warburg effect is a metabolic process where cancer cells produce energy through increased aerobic glycolysis, rather through oxidative phosphorylation (Warburg, 1925; Kang et al., 2023) The metabolite profile reported in Table 2 for the SDX group reflects this aerobic glycolysis (Bose et al., 2021), where the implanted spheroids induced significant modifications in the glycolysis and the TCA cycles. This is primarily attributed to the significant increase in serum glutamine concentration, an amino acid for gluconeogenesis (Bose et al., 2021) (Table 2). These metabolic alterations impact glutamine’s role in energy and carbon metabolism, a characteristic also observed in patients with lung cancer (Smith et al., 2016). Moreover, the elevation in pyruvic acid (Table 2), resulting from lactate metabolism, is associated with cancer angiogenesis (Guo et al., 2021)—supported by vascularisation seen in Fig. 1B. Additionally, 3-Hydroxyisobutyric acid is identified as a ketone body upregulated in non-small lung cancer patients, while creatine, when converted into phosphocreatine by cancer cells, functions as an energy reservoir (Wang et al., 2021).

Consequently, the observed rise in creatine levels in NSCLC patients, compared to healthy individuals and across different disease stages, might be associated with the heightened metabolic activity characteristic of this cancerous process (Xia et al., 2021). Inosine is a biomarker metabolite for metastasis and tumour progression (Li et al., 2021) and was detected only in the SDX animals (Table 2). This result was also reported in an A549 cell-derived xenograft, where no inosine was seen in the control animals (Xu et al., 2016). The metabolic profile of the SDX group aligns with the known metabolism of non-small cell lung carcinoma (NSCLC) cells (Hu et al., 2020). Thus, the changes in the serum metabolite profile can be attributed to the implanted cancer. It is crucial for preclinical disease models to emulate biomarkers found in human disease conditions. This is essential for increased translatability to humans and the effectiveness of the drug development pipeline, linking preclinical and clinical stages. Therefore, improving and validating preclinical models becomes imperative (Yan et al., 2020).

## Conclusion

This pilot study has shown that an implanted lung cancer spheroid can change the serum metabolites and cancer biomarkers in athymic mice. It successfully mimicked cancer biomarkers and reflected cancer metabolism. Thus, this model can potentially be used to study early cancer metabolism and pharmacometabolomics.

## Data Availability

No datasets were generated or analysed during the current study.
